# Discordant Antitumor Efficacy with Compromised Survival in High-Risk Unresectable Hepatocellular Carcinoma

**DOI:** 10.3390/cancers18182932

**Published:** 2026-09-10

**Authors:** Young Eun Seo, Chan Joong Bae, Won Jae Lee, Sun Young Chun, Jun Sik Yoon, Yang-Hyun Baek, Yeo Wool Kang, Woo Sun Rou, Jeong Eun Song, Joonho Jeong, Young Mi Hong, Sang Youn Hwang, Hyun Chin Cho, Jung Pyo Hong, Hyun Young Woo, Jeong Heo, Yu Rim Lee, Soo Young Park, Won Young Tak, Jae Hyun Yoon, Sung Bum Cho

**Affiliations:** 1 Department of Internal Medicine, Chonnam National University Hospital, Chonnam National University Medical School, Gwangju 61469, Republic of Korea; 2 Department of Internal Medicine, School of Medicine, Keimyung University, Daegu 42601, Republic of Korea; 3 Department of Internal Medicine, Dong-A University College of Medicine, Busan 49315, Republic of Korea; 4 Department of Internal Medicine, Chungnam National University College of Medicine, Daejeon 34134, Republic of Korea; 5 Department of Internal Medicine, Daegu Catholic University Medical Center, School of Medicine, Catholic University of Daegu, Daegu 42472, Republic of Korea; 6 Department of Internal Medicine, Ulsan University Hospital, University of Ulsan College of Medicine, Ulsan 44033, Republic of Korea; 7 Department of Internal Medicine, College of Medicine, Pusan National University Yangsan Hospital, Pusan National University, Yangsan 50615, Republic of Korea; 8 Department of Internal Medicine, Dongnam Institute of Radiologic & Medical Sciences, Busan 46033, Republic of Korea; 9 Department of Internal Medicine, Institute of Medical Science, College of Medicine, Gyeongsang National University, Jinju 52725, Republic of Korea; 10 Department of Internal Medicine, Biomedical Research Institute, Pusan National University Hospital, College of Medicine, Pusan National University, Busan 49241, Republic of Korea; 11 Department of Internal Medicine, School of Medicine, Kyungpook National University Hospital, Kyungpook National University, Daegu 41944, Republic of Korea

**Keywords:** hepatocellular carcinoma, atezolizumab, bevacizumab, high-risk features, duration of response, propensity score

## Abstract

Background/Objectives: Real-world data in high-risk patients remain limited; this study evaluated survival, safety, and prognostic factors in this patient group. Methods: This multicenter study retrospectively reviewed 783 patients who received Ate/Bev from 2022–2024 across South Korea. High-risk patients (*n* = 252, 32.2%) were defined by main portal vein tumor thrombus (Vp4), tumor occupancy ≥50% of liver volume, or bile duct invasion. Propensity score matching (PSM) was performed to balance host-related factors between the high-risk and non-high-risk (*n* = 531) groups. Results: After PSM (251 pairs, *n* = 502), overall survival (median 10.4 vs. 14.9 months; HR 1.57, 95% CI 1.28–1.92, *p* < 0.001) and progression-free survival (median 6.0 vs. 6.8 months; HR 1.29, 95% CI 1.08–1.53, *p* = 0.009) remained significantly worse in the high-risk group. Objective response (25.1% vs. 19.9%, *p* = 0.200) and disease control rates (71.7% vs. 76.5%, *p* = 0.262) were comparable. Among 103 responders with documented response-onset dates, an exploratory complete-case analysis showed a numerically shorter duration of response in the high-risk group, although the difference did not reach statistical significance (median, 7.1 vs. 11.3 months; HR, 1.57; 95% CI, 0.97–2.55; *p* = 0.065). Conclusions: Ate/Bev achieved similar antitumor response rates in high-risk and non-high-risk patients, but survival remained poorer in the high-risk group. High-risk status therefore did not preclude antitumor activity with Ate/Bev.

## Simple Summary

Patients with unresectable hepatocellular carcinoma who have main portal vein tumor thrombus, tumor occupancy ≥50% of liver volume, or bile duct invasion often have poor outcomes. In 783 patients from nine Korean centers, the initial rate of tumor shrinkage or control was similar between groups after matching of baseline patient factors, while survival remained poorer in the high-risk group. Among patients whose tumors responded, an exploratory analysis suggested that responses lasted for a shorter time in the high-risk group, although the difference was not statistically significant.

## 1. Introduction

In hepatocellular carcinoma (HCC), many patients are not candidates for curative-intent therapy at the time systemic treatment is considered, either because the disease is initially unresectable or because recurrence has progressed beyond the role of surgery, transplantation, or ablation [1,2,3]. In these patients, systemic therapy is the main treatment option, and therapeutic choices have expanded substantially in recent years [4,5].

The treatment paradigm for unresectable HCC changed after IMbrave150, in which atezolizumab plus bevacizumab (Ate/Bev) improved overall survival (OS) and progression-free survival (PFS) compared with sorafenib and became widely adopted as a preferred first-line regimen [6,7]. Subsequent real-world studies have reported its effectiveness and manageable safety profile in larger and more heterogeneous patient populations [8,9,10,11,12]. However, clinical outcomes remain variable, particularly among patients with extensive tumor burden or anatomically invasive disease.

An exploratory analysis of IMbrave150 identified Vp4 portal vein tumor thrombus, tumor involvement of >50% of the liver, and bile duct invasion as high-risk features associated with poor prognosis. Although Ate/Bev retained clinical benefit over sorafenib in these patients, their absolute survival outcomes were unfavorable, and bleeding events were more frequent [13]. A subsequent multicenter real-world study also reported worse survival in high-risk patients compared with non-high-risk patients, despite similar tumor response rates between the two groups [14]. Therefore, assessing post-response disease control in a real-world cohort may provide additional descriptive information alongside survival and response outcomes in this population.

The association between high-risk tumor features and clinical outcomes may also be influenced by baseline liver function and other established prognostic factors, including the albumin–bilirubin (ALBI) grade, alpha-fetoprotein (AFP) levels, and the neutrophil-to-lymphocyte ratio (NLR) [15,16,17,18,19,20]. In addition, the safety of Ate/Bev in this vulnerable population remains incompletely defined. Previous studies have reported a higher risk of gastrointestinal bleeding in high-risk patients, whereas adverse event (AE)-related treatment discontinuation rates were similar between high-risk and non-high-risk groups [13,14].

This multicenter real-world study compared survival, radiologic response, and safety according to IMbrave150-defined high-risk status. We additionally performed treatment-naive and tumor-adjusted sensitivity analyses and treated response duration as exploratory.

## 2. Materials and Methods

### 2.1. Study Population

**Figure 1:**
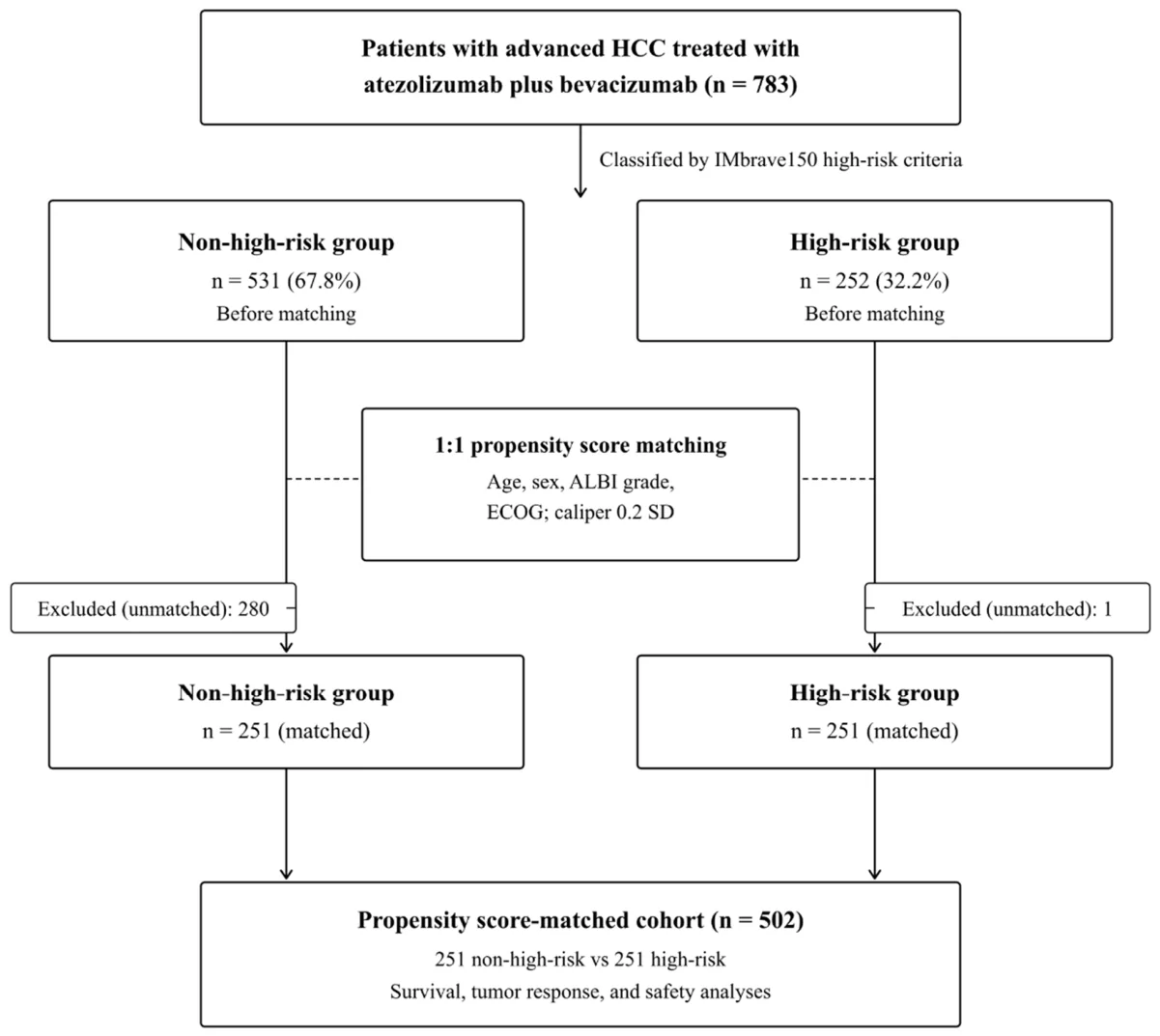
Study population.

Between January 2022 and December 2024, 783 patients with unresectable HCC who received Ate/Bev in 9 centers across South Korea were included. Of these 783 enrolled patients, 252 (32.2%) were classified as high-risk patients because they had at least one IMbrave150-defined high-risk feature: Vp4 portal vein tumor thrombus, tumor occupancy involving ≥50% of the liver volume, or bile duct invasion. The remaining 531 patients (67.8%) were classified as non-high risk [13,14]. The data cutoff was 31 August 2025. The patient selection process and overall study flow are shown in Figure 1. The study protocol was approved by the institutional review board of each participating center, including the Institutional Review Board of Chonnam National University Hwasun Hospital (IRB No. CNUHH-2024-222). The requirement for written informed consent was waived because of the retrospective nature of the study. The study was conducted in accordance with the tenets of the Declaration of Helsinki.

### 2.2. HCC Diagnosis and Baseline Assessment

HCC was diagnosed histologically and/or noninvasively based on characteristic dynamic CT or MRI findings according to an international guideline [21]. Baseline demographics, liver-disease etiology, cirrhosis, comorbidities, selected concomitant medication indicators, laboratory values including platelet count, and tumor characteristics were collected at Ate/Bev initiation. Detailed distributions are reported in Table 1; antiplatelet status was unknown in 3 patients. Prior HCC-directed treatment was recorded in 424 patients and could include overlapping modalities: TACE (*n* = 404), radiotherapy (*n* = 253), surgery (*n* = 183), radiofrequency/microwave ablation (*n* = 159), and radioembolization (*n* = 19). Liver function was assessed using ALBI grade [15,16]. Tumor characteristics, including macrovascular invasion, extrahepatic spread, tumor occupancy, Vp4, and bile duct invasion, were assessed locally on baseline CT or MRI [22].

### 2.3. Response and Adverse Event Assessment

Tumor response was assessed locally according to RECIST v1.1 using follow-up CT or MRI obtained in routine practice; imaging intervals were not protocolized and varied across centers. ORR comprised complete or partial response and DCR additionally included stable disease; patients without evaluable post-baseline imaging were counted as nonresponders. AEs were graded according to CTCAE v5.0 [23]. Duration of response (DoR) was an exploratory responder-only endpoint defined from the first documented objective response to progression or death. The primary DoR analysis was a complete-case analysis restricted to the 103 responders with a reliable documented response-onset date. In a sensitivity analysis, 10 additional responders without a reliable documented response-onset date were retained, and their response onset was assigned as 12 weeks after treatment initiation; the assigned onset was further varied from 8 to 16 weeks. Given the retrospective determination of response onset and restriction of the analysis to responders, the DoR analysis was considered exploratory and potentially susceptible to measurement and selection bias.

### 2.4. Statistical Analysis

Baseline variables were described according to high-risk classification. Before matching, continuous variables were compared using Student’s *t*-test or Mann–Whitney U test, as appropriate, and categorical variables using the chi-square test or Fisher’s exact test. In the PSM cohort, continuous variables were compared using paired *t*-tests or paired Wilcoxon tests, as appropriate; binary categorical variables were compared using exact McNemar tests, and multilevel categorical variables using the Stuart–Maxwell test.

Overall survival (OS) was calculated from the date of atezolizumab plus bevacizumab initiation to death from any cause or the last follow-up. Progression-free survival (PFS) was measured from treatment initiation to radiologic progression or death, whichever occurred first. Survival curves were generated using the Kaplan–Meier method and compared with the log-rank test. Hazard ratios (HRs) with 95% confidence intervals (CIs) were estimated using Cox proportional hazards regression. For OS and PFS analyses in the PSM cohort, Cox models used robust standard errors clustered by matched pair.

To reduce imbalance in host-related baseline characteristics, 1:1 nearest-neighbor PSM without replacement used a caliper of 0.2 SD of the logit propensity score and included age, sex, ALBI grade, and ECOG PS. Tumor variables were excluded from PSM to preserve the clinical high-risk phenotype contrast. Residual tumor imbalance was addressed in additional Cox models including infiltrative morphology and macrovascular invasion, with robust standard errors clustered by matched pair. Because Vp4 is a component of high-risk status and overlaps with macrovascular invasion, these adjusted estimates were interpreted cautiously.

The original matching generated 251 pairs (*n* = 502), which were used for survival, response, and safety analyses. A separate treatment-naive analysis included all 359 previously untreated patients; unadjusted models used the full subgroup and covariate-adjusted complete-case models included 336 patients. Treatment exposure was compared using Mann–Whitney and paired Wilcoxon tests. Statistical analyses were performed with R v.4.5.2 (R foundation for Statistical Computing, Vienna, Austria) and IBM SPSS Statistics v.25.0 (IBM Corp., Armonk, NY, USA).

## 3. Results 

### 3.1. Clinical Characteristics

The baseline clinical characteristics of the included patients are summarized in Table 1. The high-risk group showed less favorable hepatic reserve, reflected by a higher proportion of ALBI grade 2 compared with the non-high-risk group (59.5% vs. 37.1%, *p* < 0.001). The high-risk group also had a greater tumor burden, including a higher proportion of infiltrative tumor type (44.4% vs. 19.0%, *p* < 0.001) and macrovascular invasion (65.9% vs. 30.5%, *p* < 0.001). As expected from the group definition, Vp4 portal vein tumor thrombus, tumor occupancy ≥50% of liver volume, and bile duct invasion were observed only in the high-risk group. Elevated AFP levels (≥400 ng/mL; 46.3% vs. 33.8%, *p* = 0.001) and the NLR (≥3.0; 56.5% vs. 39.5%, *p* < 0.001) were also more frequent in the high-risk group. Missing data were infrequent (AFP 2.8%, NLR 2.6%). Baseline characteristics in Table 1 are presented using observed data. Prior HCC-directed treatment was less frequent in the high-risk group (35.3% vs. 63.1%). Table 1 additionally reports liver-disease etiology, cirrhosis, hypertension, diabetes, thyroid disease, and concomitant antiplatelet and anticoagulant therapy.

### 3.2. Propensity Score Matching

PSM was performed using host-related baseline factors (age, sex, ALBI grade, and ECOG PS), yielding 251 matched pairs (*n* = 502). Covariate balance before and after matching is presented in Table 1. After matching, the host factors used for matching were well balanced (all SMD < 0.10), whereas tumor-related high-risk features were intentionally left unmatched and therefore remained different between groups by design. Residual differences in infiltrative morphology and macrovascular invasion were addressed in additional Cox models.

### 3.3. Survival Outcome by Risk Group

OS and PFS according to the risk group in the full and PSM cohorts are summarized in Table 2. In the full cohort, Kaplan–Meier curves demonstrated significantly inferior OS and PFS in the high-risk group compared with the non-high-risk group (Supplementary Figure S2). The high-risk group had shorter OS (median 10.1 vs. 17.5 months; *p* < 0.001) and PFS (median 5.9 vs. 7.6 months; *p* < 0.001).

**Figure 2:**
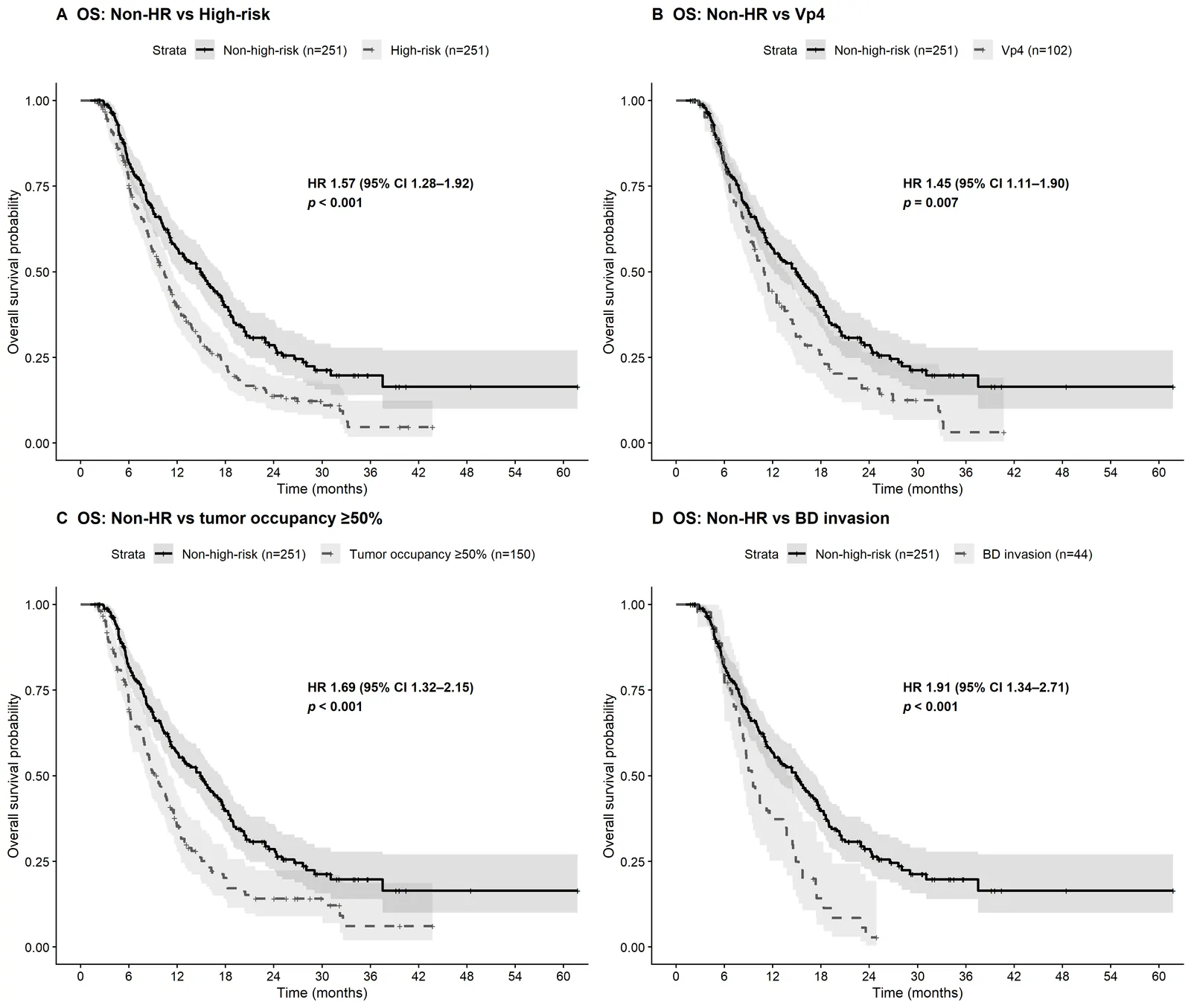
Kaplan–Meier curves for overall survival in the propensity score-matched cohort (*n* = 502). (**A**) Non-high-risk versus high-risk patients. (**B**) Non-high-risk versus patients with main portal vein tumor thrombus (Vp4). (**C**) Non-high-risk versus patients with tumor occupancy ≥50% of liver volume. (**D**) Non-high-risk versus patients with bile duct invasion.

**Figure 3:**
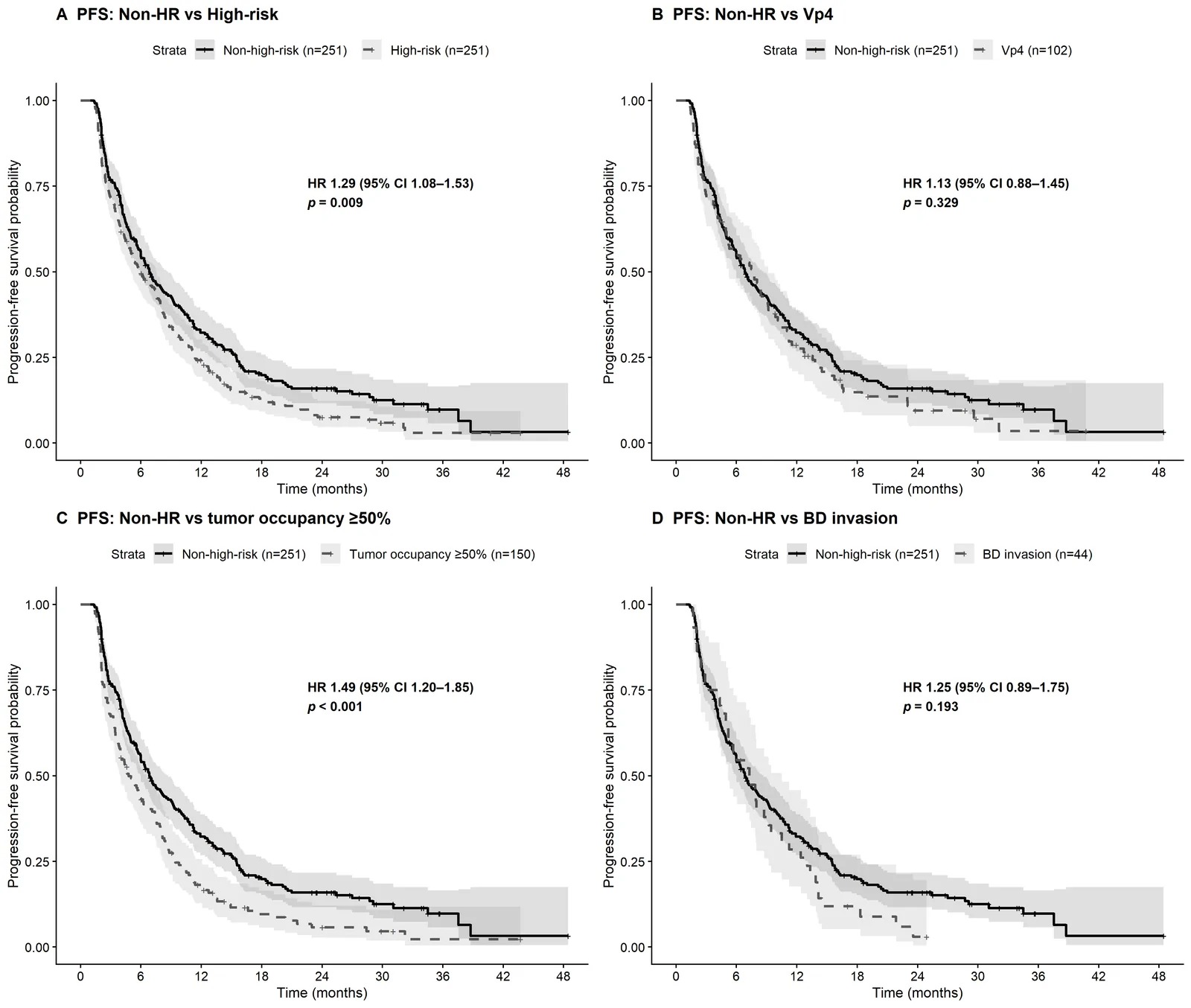
Kaplan–Meier curves for progression-free survival in the propensity score-matched cohort (*n* = 502). (**A**) Non-high-risk versus high-risk patients. (**B**) Non-high-risk versus patients with main portal vein tumor thrombus (Vp4). (**C**) Non-high-risk versus patients with tumor occupancy ≥50% of liver volume. (**D**) Non-high-risk versus patients with bile duct invasion. BD, bile duct; HR, hazard ratio; CI, confidence interval; PFS, progression-free survival.

After PSM, both OS and PFS remained significantly worse in the high-risk group (Figure 2 and Figure 3). The median OS was 10.4 months in the high-risk group versus 14.9 months in the non-high-risk group (HR 1.57, 95% CI 1.28–1.92, *p* < 0.001). The median PFS was also shorter in the high-risk group (6.0 vs. 6.8 months; HR 1.29, 95% CI 1.08–1.53, *p* = 0.009). The survival difference persisted after matching, indicating that high-risk features were associated with poorer outcomes beyond host-related baseline factors. Among 359 treatment-naive patients, high-risk status was also associated with worse OS (unadjusted HR 2.27; adjusted HR 1.93; both *p* < 0.001) and PFS (unadjusted HR 1.71; adjusted HR 1.65; both *p* < 0.001).

**Table 2:** Survival outcomes and tumor response according to high-risk classification in the full and PSM cohorts.

	Full Cohort (*n* = 783)			PSM Cohort (*n* = 502)			
	Non-HR (*n* = 531)	High Risk (*n* = 252)	*p*-Value	Non-HR (*n* = 251)	High Risk (*n* = 251)	*p*-Value	HR (95% CI)
**Survival outcomes**							
OS, median months (95% CI)	17.5 (16.2–19.0)	10.1 (9.0–11.5)	**<0.001**	14.9 (12.2–17.4)	10.4 (9.0–11.5)	**<0.001**	1.57 (1.28–1.92)
PFS, median months (95% CI)	7.6 (6.7–9.1)	5.9 (5.0–7.5)	**<0.001**	6.8 (6.0–8.7)	6.0 (5.0–7.5)	**0.009**	1.29 (1.08–1.53)
**Tumor response**							
ORR, *n* (%)	137 (25.8%)	63 (25.0%)	0.879	50 (19.9%)	63 (25.1%)	0.200	—
DCR, *n* (%)	401 (75.5%)	180 (71.4%)	0.257	192 (76.5%)	180 (71.7%)	0.262	—

OS, overall survival; PFS, progression-free survival; ORR, objective response rate; DCR, disease control rate. Survival *p*-values were obtained using the log-rank test. Full-cohort response rates were compared using Pearson’s chi-square test, whereas matched response rates were compared using the exact McNemar test. Hazard ratios for OS and PFS in the PSM cohort were estimated using univariable Cox models with robust standard errors clustered by matched pair. Bold values indicate statistical significance (*p* < 0.05).

### 3.4. Treatment Response

The best tumor responses according to risk group are summarized in Table 2. In the full cohort, ORR was comparable between the high-risk and non-high-risk groups (25.0% vs. 25.8%, *p* = 0.879), as was DCR (71.4% vs. 75.5%, *p* = 0.257). Similar findings were observed in the PSM cohort, with no significant differences in ORR (25.1% vs. 19.9%, *p* = 0.200) or DCR (71.7% vs. 76.5%, *p* = 0.262). These results indicate that the initial tumor response to Ate/Bev was broadly comparable regardless of high-risk status.

Among the 103 objective responders with a reliable documented response-onset date, the primary exploratory complete-case analysis showed a numerically shorter DoR in the high-risk group than in the non-high-risk group (60 and 43 patients, respectively; median, 7.1 vs. 11.3 months; HR, 1.57; 95% CI, 0.97–2.55; log-rank *p* = 0.065). In a sensitivity analysis including all 113 responders, response onset was assigned as 12 weeks after treatment initiation for the 10 patients without a reliable documented onset date; median DoR was 7.8 versus 13.8 months (HR, 1.72; 95% CI, 1.08–2.74; log-rank *p* = 0.021; Supplementary Figure S3). Estimates were similar when the assigned onset was varied from 8 to 16 weeks (HR, 1.71–1.72; *p* = 0.021–0.023). Because the analysis was restricted to responders, these exploratory findings should be interpreted with caution.

### 3.5. Outcomes According to Individual High-Risk Features

We further compared outcomes for each individual high-risk feature versus the non-high-risk group in the PSM cohort (Supplementary Table S1, Figure 2 and Figure 3). Tumor occupancy ≥50% of liver volume showed the most consistent association with poor outcomes, with significantly shorter OS (HR 1.69, 95% CI 1.32–2.15, *p* < 0.001) and PFS (HR 1.49, 95% CI 1.20–1.85, *p* < 0.001). Bile duct invasion was associated with worse OS (HR 1.91, 95% CI 1.34–2.71, *p* < 0.001) but not PFS (HR 1.25, *p* = 0.193), and Vp4 was associated with worse OS (HR 1.45, 95% CI 1.11–1.90, *p* = 0.007) but not PFS (HR 1.13, *p* = 0.329). ORR and DCR did not differ significantly for any individual feature. Consistent with a cumulative effect, survival worsened as the number of high-risk features increased: compared with patients having no high-risk feature, the OS HR was 1.49 (95% CI 1.20–1.86) for one feature and 2.02 (95% CI 1.46–2.78) for two or more features (Supplementary Table S2 and Supplementary Figure S1). Subgroup analyses for OS and PFS according to baseline clinical variables are provided in Supplementary Table S3; no significant effect modification was observed (for all, *p*-interaction > 0.05).

### 3.6. Prognostic Factors for Survival

In the PSM cohort, univariable Cox regression for PFS identified ALBI grade 2, ECOG PS 2, macrovascular invasion, AFP ≥ 400 ng/mL, NLR ≥ 3.0, and tumor occupancy ≥50% of liver volume as factors associated with shorter PFS. In the multivariable model, ECOG PS 2 (HR 1.56, 95% CI 1.11–2.20, *p* = 0.011), AFP ≥ 400 ng/mL (HR 1.49, 95% CI 1.21–1.84, *p* < 0.001), and tumor occupancy ≥50% of liver volume (HR 1.30, 95% CI 1.06–1.60, *p* = 0.011) remained independently associated with shorter PFS (Supplementary Table S4). In additional matched-pair Cox models adjusting for infiltrative morphology and any macrovascular invasion, high-risk status remained associated with OS (HR 1.36, 95% CI 1.09–1.70; *p* = 0.006) and PFS (HR 1.24, 95% CI 1.03–1.50; *p* = 0.025). Because Vp4, a component of high-risk status, is also a form of macrovascular invasion, these adjusted estimates should be interpreted cautiously because of potential over-adjustment.

In the PSM cohort, univariable Cox regression showed that ALBI grade 2, infiltrative tumor morphology, macrovascular invasion, AFP ≥ 400 ng/mL, NLR ≥ 3.0, tumor occupancy ≥50% of liver volume, and bile duct invasion were associated with shorter OS. In the multivariable model, ALBI grade 2 (HR 1.49, 95% CI 1.19–1.86), infiltrative morphology (HR 1.30, 95% CI 1.01–1.66), macrovascular invasion (HR 1.31, 95% CI 1.03–1.66), AFP ≥ 400 ng/mL (HR 1.57, 95% CI 1.24–2.00), tumor occupancy ≥50% of liver volume (HR 1.37, 95% CI 1.07–1.76), and bile duct invasion (HR 1.67, 95% CI 1.27–2.18) remained independently associated with shorter OS (Table 3). NLR ≥ 3.0 was significant on univariable but not multivariable analysis (adjusted HR 1.10, *p* = 0.390).

### 3.7. Safety Profiles

Safety outcomes in the full and PSM cohorts are summarized in Table 4. In the full cohort, grade ≥3 AEs were more frequent in the high-risk group than in the non-high-risk group (24.2% vs. 17.1%, *p* = 0.025), as were temporary (11.9% vs. 5.8%, *p* = 0.004) and permanent (12.7% vs. 7.9%, *p* = 0.041) treatment discontinuation. Variceal bleeding was also more frequent in the high-risk group (9.9% vs. 4.1%, *p* = 0.003), whereas severe proteinuria was less frequent (12.7% vs. 20.5%, *p* = 0.010). The median number of treatment cycles was lower in high-risk patients (7 [IQR, 3–12] vs. 9 [IQR, 4–18] cycles; *p* = 0.001).

After PSM, grade ≥3 AEs, variceal bleeding, and AE-related permanent treatment discontinuation no longer differed significantly between groups on the McNemar test (grade ≥3 AEs 24.3% vs. 18.7%, *p* = 0.156; permanent discontinuation 12.7% vs. 10.0%, *p* = 0.189; variceal bleeding 10.0% vs. 6.8%, *p* = 0.256). After PSM, the median number cycles remained lower in the high-risk group (7 [IQR 3–12] vs. 8 [IQR 4–15.5] cycles; paired *p* = 0.008).

The progression of HCC was the most common reason for discontinuation of treatment in both groups and was numerically more frequent in the high-risk group, although the difference was not statistically significant. In the full cohort, discontinuation due to liver dysfunction was more frequent in the high-risk group (15.5% vs. 8.7%, *p* = 0.006). After matching, this difference was smaller and no longer significant (16.3% vs. 12.4%, *p* = 0.237). In the matched cohort, discontinuation due to non-liver-related AEs was less frequent in the high-risk group (8.0% vs. 13.9%, *p* = 0.036). This lower frequency should be interpreted in the context of shorter treatment exposure and should not be taken as evidence of better tolerability. No significant differences were observed in loss to follow-up or other reasons for discontinuation.

## 4. Discussion

Despite the established efficacy of Ate/Bev in patients with unresectable HCC, high-risk patients have consistently shown poorer survival outcomes than non-high-risk patients. Both the IMbrave150 subgroup analysis [13] and a later real-world study reported poorer survival in high-risk patients, even though response rates were similar to non-high-risk patients [14]. We therefore evaluated initial tumor response, response durability, and survival as distinct outcomes in a multicenter real-world cohort.

The main finding is that comparable observed response rates in high-risk patients did not translate into comparable survival. Prior real-world and exploratory IMbrave150 analyses have associated durable or deeper response with longer survival [24,25]. In the present study, DoR was a secondary exploratory analysis restricted to responders. The primary complete-case analysis showed a numerically shorter DoR in the high-risk group without statistical significance, whereas the 12-week sensitivity analysis showed a statistically significant difference. Both findings should be interpreted cautiously.

Several tumor- and liver-related factors provide clinical context for this pattern. In patients with tumor occupancy of ≥50% of liver volume, even a similar degree of tumor shrinkage may leave a large amount of viable tumor. Intratumoral heterogeneity is common in HCC and may allow some tumor cells to survive treatment [26]. The association of tumor occupancy of ≥50% with both worse OS and PFS in our study is consistent with the IMbrave150 subgroup analysis [13].

Bile duct invasion and macrovascular invasion may affect disease control through both tumor progression and deterioration of liver function. Bile duct invasion can cause biliary obstruction and cholangitis, whereas portal vein tumor thrombosis may accelerate intrahepatic spread and worsen portal hypertension [27,28,29]. Immunoregulatory myeloid cells have been reported in portal vein tumor thrombi [30]. HCC also develops in a tolerogenic microenvironment in which Foxp3-positive regulatory T cells suppress antitumor immunity and may also promote pathological angiogenesis, providing a biological rationale for combining immune checkpoint inhibition with VEGF blockade [31].

In the multivariable analysis, both tumor-related and liver-related factors remained independently associated with shorter OS, including ALBI grade, infiltrative tumor morphology, macrovascular invasion, AFP ≥ 400 ng/mL, tumor occupancy of ≥50% of liver volume, and bile duct invasion. An AFP level of ≥400 ng/mL may reflect aggressive tumor biology [17], whereas a poor ALBI grade indicates impaired liver function and reduced hepatic reserve [15,16].

Safety findings should be interpreted in the context of treatment exposure. High-risk patients received fewer cycles both before and after matching; therefore, crude cumulative AE and discontinuation proportions require cautious interpretation. The lower non-liver-related AE discontinuation rate should not be interpreted as better tolerability.

Several limitations warrant consideration. The retrospective design permits residual confounding. PSM balanced host factors but intentionally retained tumor differences; additional Cox adjustment reduces but cannot eliminate this limitation, and macrovascular invasion overlaps with the Vp4 component of high-risk status. Treatment-naive and previously treated patients were mixed in the primary cohort, although a separate treatment-naive analysis supported the findings. Imaging schedules varied across centers and may have shifted recorded progression dates. Response was assessed locally without blinded central radiological review, creating potential interobserver variation in RECIST measurements and high-risk feature classification. The exploratory DoR analysis was restricted to responders, and the 12-week sensitivity analysis required assignment of response-onset dates for patients without reliable documented response-onset dates, introducing potential selection and measurement bias. Safety comparisons were limited by differences in treatment exposure. Finally, small Vp4 and bile duct invasion subgroups limited precision.

High-risk status alone should not be interpreted as evidence of absent antitumor activity with Ate/Bev. Similar response rates nevertheless did not translate into comparable survival. Further prospective studies are warranted to better define treatment outcomes in this high-risk population.

## 5. Conclusions

In this multicenter real-world study, Ate/Bev demonstrated similar objective response rates in high-risk and non-high-risk patients with HCC, whereas OS and PFS remained poorer in the high-risk group. In the exploratory DoR analysis, the primary complete-case analysis showed a numerically shorter DoR in high-risk responders without statistical significance, while the 12-week sensitivity analysis showed a statistically significant difference. These findings suggest that high-risk status does not preclude antitumor activity with Ate/Bev but remains associated with poorer survival outcomes. The DoR findings should be interpreted cautiously.

## Figures and Tables

**Table 1:** Baseline characteristics before and after propensity score matching.

	Before PSM					After PSM			
	Overall (*n* = 783)	Non-HR (*n* = 531)	High Risk (*n* = 252)	*p*	SMD	Non-HR (*n* = 251)	High Risk (*n* = 251)	*p*	SMD
Age, years, mean ± SD	65.72 ± 9.71	66.11 ± 9.39	64.88 ± 10.34	0.111	0.124	65.24 ± 9.58	64.91 ± 10.35	0.536	0.032
Sex, *n* (%)				0.086	0.129			1.000	0.010
Male	665 (84.9%)	459 (86.4%)	206 (81.7%)			205 (81.7%)	206 (82.1%)		
Female	118 (15.1%)	72 (13.6%)	46 (18.3%)			46 (18.3%)	45 (17.9%)		
Etiology, *n* (%)				0.679	0.089			0.492	0.121
HBV	467 (59.6%)	312 (58.8%)	155 (61.5%)			139 (55.4%)	154 (61.4%)		
HCV	75 (9.6%)	54 (10.2%)	21 (8.3%)			27 (10.8%)	21 (8.4%)		
HBV+HCV	18 (2.3%)	14 (2.6%)	4 (1.6%)			8 (3.2%)	4 (1.6%)		
Alcohol	152 (19.4%)	101 (19.0%)	51 (20.2%)			50 (19.9%)	51 (20.3%)		
MASLD	6 (0.8%)	4 (0.8%)	2 (0.8%)			2 (0.8%)	2 (0.8%)		
Cryptogenic	64 (8.2%)	46 (8.7%)	18 (7.1%)			25 (10.0%)	18 (7.2%)		
Mixed HCV/alcohol	1 (0.1%)	0 (0.0%)	1 (0.4%)			0 (0.0%)	1 (0.4%)		
Cirrhosis, *n* (%)	666 (85.1%)	447 (84.2%)	219 (86.9%)	0.318	0.078	217 (86.5%)	218 (86.9%)	1.000	0.012
Hypertension, *n* (%)	330 (42.1%)	237 (44.6%)	93 (36.9%)	**0.041**	0.158	115 (45.8%)	93 (37.1%)	**0.047**	0.179
Diabetes, *n* (%)	265 (33.8%)	190 (35.8%)	75 (29.8%)	0.096	0.129	91 (36.3%)	75 (29.9%)	0.152	0.136
Antiplatelet therapy, *n* (%)	64 (8.2%)	40 (7.6%)	24 (9.5%)	0.354	0.070	17 (6.8%)	24 (9.6%)	0.337	0.101
Anticoagulant therapy, *n* (%)	49 (6.3%)	34 (6.4%)	15 (6.0%)	0.808	0.019	19 (7.6%)	15 (6.0%)	0.597	0.063
Thyroid disease, *n* (%)	24 (3.1%)	17 (3.2%)	7 (2.8%)	0.748	0.025	7 (2.8%)	7 (2.8%)	1.000	0.000
ALBI grade, *n* (%)				**<0.001**	0.460			1.000	0.000
Grade 1	436 (55.7%)	334 (62.9%)	102 (40.5%)			102 (40.6%)	102 (40.6%)		
Grade 2	347 (44.3%)	197 (37.1%)	150 (59.5%)			149 (59.4%)	149 (59.4%)		
ECOG performance status, *n* (%)				0.579	0.049			0.500	0.064
0–1	768 (98.1%)	522 (98.3%)	246 (97.6%)			248 (98.8%)	246 (98.0%)		
2	15 (1.9%)	9 (1.7%)	6 (2.4%)			3 (1.2%)	5 (2.0%)		
BCLC stage, *n* (%)				0.161	0.110			0.081	0.173
A/B	93 (11.9%)	69 (13.0%)	24 (9.5%)			37 (14.7%)	23 (9.2%)		
C	690 (88.1%)	462 (87.0%)	228 (90.5%)			214 (85.3%)	228 (90.8%)		
Tumor morphology, *n* (%)				**<0.001**	0.568			<0.001	0.487
Non-infiltrative	570 (72.8%)	430 (81.0%)	140 (55.6%)			195 (77.7%)	139 (55.4%)		
Infiltrative	213 (27.2%)	101 (19.0%)	112 (44.4%)			56 (22.3%)	112 (44.6%)		
Macrovascular invasion, *n* (%)				**<0.001**	0.757			<0.001	0.663
Absent	455 (58.1%)	369 (69.5%)	86 (34.1%)			164 (65.3%)	85 (33.9%)		
Present	328 (41.9%)	162 (30.5%)	166 (65.9%)			87 (34.7%)	166 (66.1%)		
Extrahepatic spread, *n* (%)				**0.001**	0.246			0.069	0.177
Absent	324 (41.4%)	199 (37.5%)	125 (49.6%)			102 (40.6%)	124 (49.4%)		
Present	459 (58.6%)	332 (62.5%)	127 (50.4%)			149 (59.4%)	127 (50.6%)		
AFP, *n* (%)				**0.001**	0.256			0.008	0.240
<400 ng/mL	473 (62.2%)	342 (66.2%)	131 (53.7%)			162 (65.6%)	131 (53.9%)		
≥400 ng/mL	288 (37.8%)	175 (33.8%)	113 (46.3%)			85 (34.4%)	112 (46.1%)		
NLR, *n* (%)				**<0.001**	0.346			0.005	0.256
<3.0	420 (55.0%)	313 (60.5%)	107 (43.5%)			137 (56.4%)	107 (43.7%)		
≥3.0	343 (45.0%)	204 (39.5%)	139 (56.5%)			106 (43.6%)	138 (56.3%)		
Prior treatment, *n* (%)				**<0.001**	0.578			<0.001	0.528
No	359 (45.8%)	196 (36.9%)	163 (64.7%)			98 (39.0%)	162 (64.5%)		
Yes	424 (54.2%)	335 (63.1%)	89 (35.3%)			153 (61.0%)	89 (35.5%)		
Vp4 portal vein invasion, *n* (%)									
No	681 (87.0%)	531 (100.0%)	150 (59.5%)			251 (100.0%)	149 (59.4%)		
Yes	102 (13.0%)	0 (0.0%)	102 (40.5%)			0 (0.0%)	102 (40.6%)		
Tumor extent ≥50% of liver, *n* (%)									
<50%	632 (80.7%)	531 (100.0%)	101 (40.1%)			251 (100.0%)	101 (40.2%)		
≥50%	151 (19.3%)	0 (0.0%)	151 (59.9%)			0 (0.0%)	150 (59.8%)		
Bile duct invasion, *n* (%)									
No	739 (94.4%)	531 (100.0%)	208 (82.5%)			251 (100.0%)	207 (82.5%)		
Yes	44 (5.6%)	0 (0.0%)	44 (17.5%)			0 (0.0%)	44 (17.5%)		

Values are presented as mean ± SD or *n* (%). Full-cohort *p*-values were calculated using *t*-tests, uncorrected Pearson chi-square tests, or Fisher exact tests; matched-cohort *p*-values used paired *t*-tests, exact McNemar tests for binary variables, or the Stuart-Maxwell test for multilevel categorical variables. For multilevel categorical variables, the reported SMD is the maximum absolute category-specific SMD. SMD, standardized mean difference; HR, high-risk; Non-HR, non-high-risk; PSM, propensity score matching. AFP and NLR were missing in 22 and 20 patients in the full cohort and in 12 and 14 patients in the matched cohort, respectively; antiplatelet status was unknown in 3 patients in the full cohort and 1 patient in the matched cohort. Bold values indicate statistical significance (*p* < 0.05).

**Table 3:** Univariable and multivariable Cox regression analysis for overall survival (PSM cohort *n* = 502).

			Univariable		Multivariable	
	Events/ Patients	Median OS, Months (95% CI)	HR (95% CI)	*p* Value	HR (95% CI)	*p* Value
Age				0.780		—
<65	167/235	11.6 (10.3–14.3)	1.00 (ref)		—	
≥65	189/267	11.6 (10.5–13.8)	0.97 (0.79–1.20)		—	
Sex				0.516		—
Male	297/411	11.6 (10.8–13.1)	1.00 (ref)		—	
Female	59/91	11.5 (9.6–16.2)	0.90 (0.67–1.23)		—	
ALBI grade				**<0.001**		**<0.001**
Grade 1	133/204	15.0 (13.1–18.3)	1.00 (ref)		1.00 (ref)	
Grade 2	223/298	10.0 (8.8–11.2)	1.65 (1.34–2.04)		**1.49 (1.19–1.86)**	
ECOG performance status				0.087		—
0–1	349/494	11.6 (10.9–13.1)	1.00 (ref)		—	
2	7/8	10.3 (5.2–NA)	1.55 (0.94–2.57)		—	
BCLC stage				0.770		—
A/B	48/60	11.2 (10.1–15.2)	1.00 (ref)		—	
C	308/442	11.6 (10.7–13.5)	0.96 (0.73–1.27)		—	
Tumor morphology				**<0.001**		**0.041**
Non-infiltrative	227/334	12.5 (11.5–14.9)	1.00 (ref)		1.00 (ref)	
Infiltrative	129/168	9.6 (8.2–11.3)	1.46 (1.18–1.81)		**1.30 (1.01–1.66)**	
Macrovascular invasion				**<0.001**		**0.028**
Absent	158/249	13.8 (11.8–16.3)	1.00 (ref)		1.00 (ref)	
Present	198/253	10.0 (8.8–11.6)	1.57 (1.29–1.92)		**1.31 (1.03–1.66)**	
Extrahepatic spread				0.497		—
Absent	168/226	12.1 (10.8–14.4)	1.00 (ref)		—	
Present	188/276	11.4 (10.4–13.5)	0.94 (0.77–1.13)		—	
AFP				**<0.001**		**<0.001**
<400 ng/mL	194/293	14.3 (12.2–16.0)	1.00 (ref)		1.00 (ref)	
≥400 ng/mL	153/197	9.1 (8.1–10.9)	1.71 (1.36–2.13)		**1.57 (1.24–2.00)**	
NLR				**<0.001**		0.390
<3.0	158/244	14.6 (12.0–17.7)	1.00 (ref)		1.00 (ref)	
≥3.0	190/244	10.4 (8.9–11.5)	1.48 (1.21–1.82)		1.10 (0.88–1.37)	
Prior treatment				0.372		—
No	188/260	11.6 (10.4–13.8)	1.00 (ref)		—	
Yes	168/242	11.5 (10.7–14.5)	0.91 (0.74–1.12)		—	
Vp4 portal vein invasion				0.133		—
No	276/400	11.9 (10.9–13.8)	1.00 (ref)		—	
Yes	80/102	10.9 (9.1–13.5)	1.19 (0.95–1.51)		—	
Tumor extent ≥ 50%				**<0.001**		**0.013**
<50%	242/352	13.5 (11.5–15.3)	1.00 (ref)		1.00 (ref)	
≥50%	114/150	9.5 (8.1–11.3)	1.54 (1.23–1.94)		**1.37 (1.07–1.76)**	
Bile duct invasion				**0.001**		**<0.001**
No	316/458	11.9 (11.0–13.5)	1.00 (ref)		1.00 (ref)	
Yes	40/44	9.4 (8.3–14.5)	1.56 (1.20–2.03)		**1.67 (1.27–2.18)**	

HR, hazard ratio; CI, confidence interval; AFP, alpha-fetoprotein; NLR, neutrophil-to-lymphocyte ratio. Hazard ratios were estimated using Cox regression with robust standard errors clustered on the matched pair. Variables with *p* < 0.05 on univariable analysis were entered into the multivariable model. AFP and NLR were missing in 12 and 14 patients, respectively, who were excluded from the corresponding analyses (multivariable complete-case *n* = 476). — indicates a variable not included in the multivariable model. Bold values indicate statistical significance (*p* < 0.05). NA indicates that the upper limit of the 95% confidence interval for median OS was not estimable.

**Table 4:** Safety profile and reasons for chemotherapy discontinuation according to high-risk classification.

	Full Cohort (*n* = 783)			PSM Cohort (*n* = 502)		
	Non-HR (*n* = 531)	High Risk (*n* = 252)	*p*-Value	Non-HR (*n* = 251)	High Risk (*n* = 251)	*p*-Value
**Safety profile**						
Varix bleeding	22 (4.1%)	25 (9.9%)	**0.003**	17 (6.8%)	25 (10.0%)	0.256
Severe proteinuria (≥3+)	109 (20.5%)	32 (12.7%)	**0.010**	42 (16.7%)	32 (12.7%)	0.253
AE Grade 1–2	198 (37.3%)	81 (32.1%)	0.185	88 (35.1%)	80 (31.9%)	0.475
AE Grade ≥3	91 (17.1%)	61 (24.2%)	**0.025**	47 (18.7%)	61 (24.3%)	0.156
Temporary treatment discontinuation	31 (5.8%)	30 (11.9%)	**0.004**	23 (9.2%)	30 (12.0%)	0.230
Permanent treatment discontinuation	42 (7.9%)	32 (12.7%)	**0.041**	25 (10.0%)	32 (12.7%)	0.189
**Treatment exposure, median cycles (IQR)**	**9 (4–18)**	7 (3–12)	**0.001**	8 (4–15.5)	7 (3–12)	**0.008**
**Reasons for treatment discontinuation**						
HCC progression	315 (59.3%)	159 (63.1%)	0.352	141 (56.2%)	158 (62.9%)	0.128
Liver dysfunction	46 (8.7%)	39 (15.5%)	**0.006**	31 (12.4%)	41 (16.3%)	0.237
Non-liver related AE	60 (11.3%)	22 (8.7%)	0.331	35 (13.9%)	20 (8.0%)	**0.036**
F/U loss	23 (4.3%)	10 (4.0%)	0.963	9 (3.6%)	10 (4.0%)	1.000
Other ‡	26 (4.9%)	11 (4.4%)	0.883	8 (3.2%)	11 (4.4%)	0.607
Ongoing/Missing	61 (11.5%)	11 (4.4%)		27 (10.8%)	11 (4.4%)	

Values are *n* (%) unless otherwise stated. Full-cohort *p*-values use chi-square tests with continuity correction; matched binary outcomes use exact McNemar tests. Treatment exposure was compared using Mann–Whitney and paired Wilcoxon tests. AE, adverse event; IQR, interquartile range. ‡ Other includes completion of 2-year treatment, cost/insurance issues, and patient refusal. Bold values indicate statistical significance (*p* < 0.05).

## Data Availability

De-identified data may be made available from the corresponding authors upon reasonable request.

## Supplementary Materials

The following supporting information can be downloaded at: https://www.mdpi.com/article/10.3390/cancers18182932/s1, Figure S1: Survival outcomes according to cumulative high-risk feature burden in the propensity score-matched cohort; Figure S2: Kaplan–Meier curves for overall survival and progression-free survival in the full cohort (*n* = 783); Figure S3: Sensitivity analysis of duration of response among 113 responders in the propensity score-matched cohort; Table S1: Survival and tumor response outcomes by individual high-risk feature vs. non-high-risk group in the PSM cohort; Table S2: Survival outcomes according to cumulative high-risk feature burden in the propensity score-matched cohort; Table S3: Subgroup analysis for overall survival and progression-free survival in the propensity score-matched cohort (*n* = 502); Table S4: Univariable and multivariable Cox regression analysis for progression-free survival (PSM cohort, *n* = 502).

## Abbreviations

Ate+Bev	Atezolizumab plus bevacizumab
ALBI	albumin–bilirubin grade
CTCAE	Common Terminology Criteria for Adverse Events
DCR	disease control rate
NLR	neutrophil-to-lymphocyte ratio
ORR	objective response rate
OS	overall survival
PFS	progression-free survival
PS	performance status
PSM	Propensity score matching
SMD	standardized mean differences
Vp4	main portal vein tumor thrombus
