# Supplementary material for: Discordant Antitumor Efficacy with Compromised Survival in High-Risk Unresectable Hepatocellular Carcinoma
Source: Cancers (Basel). 2026 Sep 10;18(18):2932. doi: 10.3390/cancers18182932 (PMC13604502; doi:10.3390/cancers18182932)
Supplement: Supplementary file 1 [file cancers-18-02932-s001.zip › Supplementary Table S1.pdf]

Supplementary Table S1. Survival and tumor response outcomes by individual high-risk feature vs. non-high-risk group in the PSM cohort

Feature	Endpoint	Non-High-Risk (n=251)	Feature Group	HR (95% CI)	p-value
Vp4 (n=102)	OS	14.9 (12.2–17.4) mo	10.9 (9.1–13.5) mo	1.45 (1.11–1.90)	**0.007**
	PFS	6.8 (6.0–8.7) mo	7.5 (5.2–9.2) mo	1.13 (0.88–1.45)	0.329
	ORR	19.9%	27.5%	—	0.160
	DCR	76.5%	72.5%	—	0.520
Tumor occupancy ≥50% (n=150)	OS	14.9 (12.2–17.4) mo	9.5 (8.1–11.3) mo	1.69 (1.32–2.15)	**<0.001**
	PFS	6.8 (6.0–8.7) mo	4.8 (3.9–6.5) mo	1.49 (1.20–1.85)	**<0.001**
	ORR	19.9%	22.0%	—	0.711
	DCR	76.5%	68.0%	—	0.081
BD Invasion (n=44)	OS	14.9 (12.2–17.4) mo	9.4 (8.3–14.5) mo	1.91 (1.34–2.71)	**<0.001**
	PFS	6.8 (6.0–8.7) mo	7.3 (5.2–10.5) mo	1.25 (0.89–1.75)	0.193
	ORR	19.9%	27.3%	—	0.366
	DCR	76.5%	81.8%	—	0.560

Analyses based on PSM cohort. Each feature group compared to the non-high-risk group.HR from univariable Cox model. ORR/DCR p-values from chi-squared test.
