# Supplementary material for: Discordant Antitumor Efficacy with Compromised Survival in High-Risk Unresectable Hepatocellular Carcinoma
Source: Cancers (Basel). 2026 Sep 10;18(18):2932. doi: 10.3390/cancers18182932 (PMC13604502; doi:10.3390/cancers18182932)
Supplement: Supplementary file 1 [file cancers-18-02932-s001.zip › Supplementary Table S2.pdf]

Supplementary Table S2. Survival outcomes according to cumulative high-risk feature burden in the propensity score-matched cohort

**Outcome**	Cumulative high-risk Features	N	Median, months (95% CI)	HR (95% CI)	P value
**OS**	0	251	14.9 (12.2–17.4)	Reference	—
	1	207	10.7 (9.5–11.8)	1.49 (1.20–1.86)	<0.001
	≥2	44	8.8 (7.6–12.5)	2.02 (1.46–2.78)	<0.001
**PFS**	0	251	6.8 (6.0–8.7)	Reference	—
	1	207	6.0 (5.0–7.6)	1.25 (1.04–1.51)	0.020
	≥2	44	5.9 (4.3–9.1)	1.48 (1.13–1.93)	0.004

High-risk features were Vp4 portal vein invasion, tumor occupancy ≥50% of liver volume, and bile duct invasion. Patients with two or three high-risk features were combined because only one patient had all three features. Categorical hazard ratios use 0 high-risk features as the reference. Trend hazard ratios represent the effect per one additional high-risk feature. Hazard ratios were estimated using Cox proportional hazards models with robust standard errors clustered by propensity score-matched subclass. CI, confidence interval; HR, hazard ratio; OS, overall survival; PFS, progression-free survival; Vp4, portal vein tumor thrombosis involving the main trunk and/or contralateral branch.
