# Supplementary material for: Discordant Antitumor Efficacy with Compromised Survival in High-Risk Unresectable Hepatocellular Carcinoma
Source: Cancers (Basel). 2026 Sep 10;18(18):2932. doi: 10.3390/cancers18182932 (PMC13604502; doi:10.3390/cancers18182932)
Supplement: Supplementary file 1 [file cancers-18-02932-s001.zip › Supplementary Table S3.pdf]

Supplementary Table S3. Subgroup analysis for overall survival and progression-free survival in the propensity score-matched cohort (n=502)

		Overall Survival			Progression-Free Survival		
**Subgroup**	**N**	**HR (95% CI)**	**p**	**p-interaction**	**HR (95% CI)**	**p**	**p-interaction**
**Age**				0.753			0.579
<65	235	**1.63 (1.19–2.24)**	**0.002**		**1.34 (1.05–1.73)**	**0.021**	
≥65	267	**1.50 (1.16–1.96)**	**0.002**		1.23 (0.97–1.56)	0.094	
**Sex**				0.056			0.838
Male	411	**1.44 (1.15–1.81)**	**0.001**		**1.30 (1.07–1.58)**	**0.008**	
Female	91	**2.37 (1.50–3.75)**	**<0.001**		1.23 (0.86–1.78)	0.258	
**ALBI grade**				0.131			0.330
Grade 1	204	**1.94 (1.38–2.73)**	**<0.001**		**1.42 (1.07–1.89)**	**0.016**	
Grade 2	298	**1.34 (1.04–1.73)**	**0.025**		1.20 (0.96–1.49)	0.107	
**ECOG PS**				—			—
0-1	494	**1.58 (1.29–1.94)**	**<0.001**		**1.28 (1.07–1.52)**	**0.006**	
**Tumor morphology**				0.569			0.241
Non-infiltrative	187	1.43 (0.97–2.10)	0.071		1.00 (0.70–1.42)	0.986	
Infiltrative	298	1.26 (0.96–1.65)	0.091		1.29 (1.00–1.66)	0.053	
**AFP**				0.317			0.729
<400 ng/mL	334	**1.57 (1.22–2.02)**	**<0.001**		**1.30 (1.05–1.61)**	**0.015**	
≥400 ng/mL	168	1.23 (0.85–1.80)	0.274		1.22 (0.85–1.73)	0.278	
**NLR**				0.423			0.357
<3.0	293	**1.62 (1.25–2.12)**	**<0.001**		**1.32 (1.06–1.65)**	**0.015**	
≥3.0	197	1.31 (0.95–1.81)	0.098		1.08 (0.81–1.44)	0.603	
**Prior HCC-direct treatment**				0.328			0.269
No	244	**1.71 (1.25–2.34)**	**<0.001**		1.45 (1.11–1.89)	0.006	
Yes	244	**1.34 (1.01–1.80)**	**0.046**		1.13 (0.87–1.47)	0.364	
