# Supplementary material for: Discordant Antitumor Efficacy with Compromised Survival in High-Risk Unresectable Hepatocellular Carcinoma
Source: Cancers (Basel). 2026 Sep 10;18(18):2932. doi: 10.3390/cancers18182932 (PMC13604502; doi:10.3390/cancers18182932)
Supplement: Supplementary file 1 [file cancers-18-02932-s001.zip › Supplementary Table S4.pdf]

Supplementary Table S4. Univariable and multivariable Cox regression analysis for progression-free survival (PSM cohort, n=502)

			Univariable		Multivariable	
	Events/ Patients	Median PFS, months (95% CI)	HR (95% CI)	p value	HR (95% CI)	p value
Age				0.101		—
<65	208/235	5.8 (4.6–7.2)	1.00 (ref)		—	
≥65	223/267	7.0 (6.0–8.2)	0.84 (0.69–1.03)		—	
Sex				0.979		—
Male	356/411	6.8 (5.8–7.9)	1.00 (ref)		—	
Female	75/91	5.8 (4.4–8.0)	1.00 (0.74–1.36)		—	
ALBI grade				**0.040**		0.193
Grade 1	170/204	7.4 (6.0–8.8)	1.00 (ref)		1.00 (ref)	
Grade 2	261/298	5.9 (5.2–7.1)	1.25 (1.01–1.54)		1.16 (0.93–1.46)	
ECOG performance status				**0.006**		**0.011**
0-1	423/494	6.5 (5.8–7.5)	1.00 (ref)		1.00 (ref)	
2	8/8	4.5 (3.4–NA)	1.66 (1.16–2.39)		**1.56 (1.11–2.20)**	
BCLC stage				0.488		—
A/B	53/60	7.8 (5.5–11.2)	1.00 (ref)		—	
C	378/442	6.4 (5.6–7.4)	1.10 (0.84–1.44)		—	
Tumor morphology				0.504		—
Non-infiltrative	286/334	6.5 (5.6–7.6)	1.00 (ref)		—	
Infiltrative	145/168	6.4 (5.3–8.4)	1.07 (0.88–1.29)		—	
Macrovascular invasion				**0.034**		0.226
Absent	210/249	7.1 (6.0–8.5)	1.00 (ref)		1.00 (ref)	
Present	221/253	5.8 (5.0–7.2)	1.22 (1.01–1.46)		1.13 (0.93–1.36)	
Extrahepatic spread				0.099		—

Supplementary Table S4. Univariable and multivariable Cox regression analysis for progression-free survival (PSM cohort, n=502)

			Univariable		Multivariable	
	Events/ Patients	Median PFS, months (95% CI)	HR (95% CI)	p value	HR (95% CI)	p value
Absent	193/226	7.0 (5.9–8.8)	1.00 (ref)		—	
Present	238/276	6.0 (4.8–7.5)	1.16 (0.97–1.39)		—	
AFP				**<0.001**		**<0.001**
<400 ng/mL	243/293	8.2 (7.1–9.2)	1.00 (ref)		1.00 (ref)	
≥400 ng/mL	177/197	4.4 (3.9–5.5)	1.56 (1.27–1.93)		**1.49 (1.21–1.84)**	
NLR				**0.027**		0.648
<3.0	202/244	7.5 (6.0–8.8)	1.00 (ref)		1.00 (ref)	
≥3.0	216/244	6.0 (5.2–7.0)	1.23 (1.02–1.49)		1.05 (0.86–1.28)	
Prior treatment				0.334		—
No	223/260	7.1 (6.0–8.2)	1.00 (ref)		—	
Yes	208/242	5.8 (4.8–7.1)	1.10 (0.91–1.34)		—	
Vp4 portal vein invasion				0.989		—
No	341/400	6.3 (5.6–7.2)	1.00 (ref)		—	
Yes	90/102	7.5 (5.2–9.2)	1.00 (0.80–1.25)		—	
Tumor occupancy ≥50% of liver volume				**<0.001**		**0.011**
<50%	295/352	7.1 (6.3–8.3)	1.00 (ref)		1.00 (ref)	
≥50%	136/150	4.8 (3.9–6.5)	1.47 (1.22–1.79)		**1.30 (1.06–1.60)**	
Bile duct invasion				0.426		—
No	390/458	6.4 (5.7–7.5)	1.00 (ref)		—	
Yes	41/44	7.3 (5.2–10.5)	1.11 (0.86–1.45)		—	
